# Factors Associated with Occupational Injury among Hydropower Dam Construction Workers, South East Ethiopia, 2018

**DOI:** 10.1155/2020/6152612

**Published:** 2020-04-26

**Authors:** Jemal Hussen, Henok Dagne, Dawit Getachew Yenealem

**Affiliations:** Department of Environmental and Occupational Health and Safety, Institute of Public Health, College of Medicine and Health Sciences, University of Gondar, Ethiopia

## Abstract

**Background:**

Occupational injuries pose a major public health and socioeconomic developmental problems. Globally, 160 million people encounter occupational injuries; the International Labour Organization estimates that the cost is 4**%** of the global gross domestic product (GDP) or 1.25 trillion United States Dollar (USD). The second-largest number of occupational injuries was reported from the construction industries. There are limited studies about the prevalence and factors associated with occupational injuries among dam construction workers in Ethiopia. Hence, this study was undertaken to determine the prevalence and associated factors of occupational injury among Genale Dawa hydropower dam construction workers.

**Method:**

Institutional-based cross-sectional study was conducted in Genale Dawa 3D hydropower dam construction project from April 1 to 22, 2018. Four hundred and five workers were included in the study. An Oromiffa version pretested, semistructured questionnaire was used to collect data. Data were entered into Epi-info version 7, and analysis was done using SPSS version 20 software. Bivariable and multivariate binary logistic regression was used to see the association between predictors and the dependent variable. The 95% CI and adjusted odds ratio with a *P* value of 0.05 was used to fit the final model.

**Results:**

The prevalence of occupational injuries in the earlier 12 months before the study was 57.8% with (95% CI (52.8, 62.7)). Age, educational status, alcohol consumption, job stress, work shift, and working hours per week were factors significantly associated with occupational injury. *Conclusion and recommendation*. Occupational injuries were common among dam construction workers. Conducting regular monitoring of substance abuse, avoiding overtime work, rotation of the work shift, and considering age and the educational status during employee recruitment can be effective to decrease the prevalence of occupational injuries.

## 1. Background

An occupational injury is any physical injury condition on a worker associated with the performing workers at the workplace [1]. Globally, 160 million people live with work-related injuries that resulted in four days and above absence from work in each year: the International Labour Organization believes that the costs of occupational injuries and accidents varied between 1.8% and 6% of GDP among different countries, averaged at the world to be 4% of the global GDP or 1.25 trillion USD [2, 3]. Globally, construction is a dangerous industry with high rates of fatal and nonfatal injuries [4]. The prevalence of fatal and nonfatal occupational injury is 10 to 20 times higher in developing countries because of a lack of access to occupational health services [5]. Sub-Saharan Africa has the highest rate per worker of occupational injuries followed by Asia (excluding China and India) [5]. Only 5 % to 10% of the workforce in developing countries has access to some kind of occupational health and safety services [6]. Literature showed that the prevalence of occupational injuries among construction workers at different countries was 30.1% in Iran [7], 30% in Turkey [8], 46.2% in Egypt [9], 71% in Illam (West Iran) [10], 74% in Kenya [11], and the prevalence varied between 38.3% [12], 38.7% [13], and 84.7% [14] in earlier studies done in Ethiopia. In a study conducted in Addis Ababa [14] sex, job satisfaction, workload, training, and personal protective equipment use were predictors of occupational injury. Studies [1, 12–15] showed that factors contributing to occupational injuries were lack of safety training, job stress, the absence of a safety sign, a sleep problem, workload, drinking alcohol, and chewing khat. Some studies conducted on construction work in Ethiopia about occupational injuries recommended health and safety interventions to reduce occupational injuries and loss of productivity in the workplace [13, 14]. Occupational injuries and loss of productivity continued to be a major challenge in the construction industry 53 [12].

**Figure 1:**
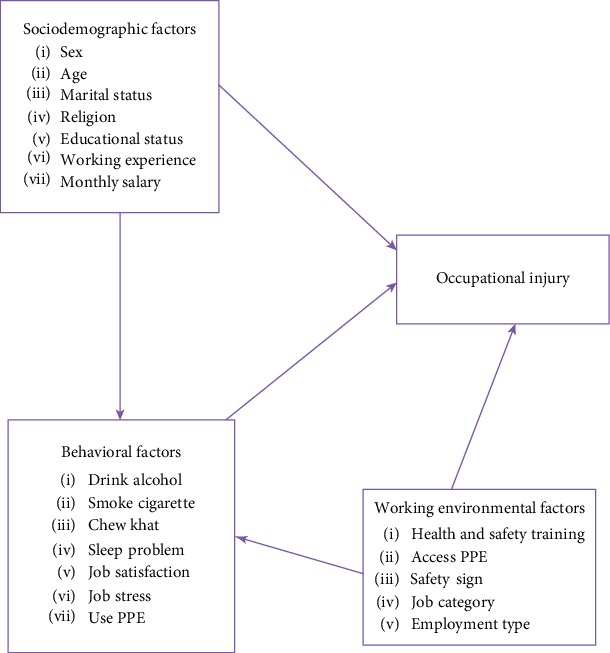
Conceptual framework adapted from the literature review [4, 12, 16].

The existing few studies [12–14] conducted on occupational injuries among construction workers focused only on building construction. An extensive literature search by the authors did not show any study about the occupational injury on workers working in the hydropower dam construction and did not discuss factors, such as work shift. To make the factors associated with occupational injury clearly visible and to create better understanding, we designed a conceptual framework based on a literature review in which factors and response variables reside as interdependent (Figure 1). The aim of the present study was to assess the prevalence and factors associated with occupational injury among Genale Dawa 3D hydropower dam construction workers.

## 2. Methods

### 2.1. Study Design

The cross-sectional study design was used to assess the prevalence and factors associated with occupational injury among Genale Dawa 3D hydropower dam construction workers from April 1 to 22, 2018.

### 2.2. Study Setting

The Genale Dawa 3D hydropower dam construction project is owned by the Ethiopian government at Guji Zone, one of the 18 zones in Oromia Regional State, 640 km from Addis Ababa (capital of Ethiopia). Construction of the dam was started in 2014 and is being constructed by the Chinese Corporation named China Guzeuba Group Company (CGGC). The dam can hold 2.57 billion cubic meters of water, and it is expected to generate 254 MW electricity. Six hundred and sixty-eight employees take part in construction work.

### 2.3. Participants

The source population was all Genale Dawa 3D hydropower dam construction project workers who were involved in construction work. Of these, those who had at least one-year work experience and who were selected by a simple random sampling technique were included. Workers who were seriously ill (unable to respond due to the illnesses other than occupational injury), administrative workers who did not participate in the construction operation, and workers absent from work for any reason during the data collection period were excluded from the study.

### 2.4. Sampling Technique and Procedures

Single population proportion formula was used to determine the sample size for the prevalence of occupational injury with the following assumptions: p (prevalence of occupational injury among construction work 38.7% [13] from a study conducted in Gondar, Ethiopia), 95% confidence interval, and 5% margin of error (d). The sample size for the associated factors was calculated by using Epi-info, based on the following assumptions: the proportion of unexposed group (*P*1), the proportion of an exposed group (*P*2), adjusted odds ratio (*AOR*), the ratio of unexposed to exposed (*R*), and power 80% (*β* = the probability of rejecting a true difference 20%).

(1) $$\begin{array}{c} n=\frac{{\left(Z_{a/2}\right)}^{2}p\left(1-p\right)}{d^{2}}=\frac{{\left(1.96\right)}^{2}0.387\left(1-0.387\right)}{0.05^{2}}=365 \end{array}$$

Taking the largest sample size and adding 5% for the nonresponse rate, the total sample size was 412 workers. The study populations were stratified into four different strata: daily labourer, carpenter/mason, welder/electrician/technician, and driver/operator. Assuming that factors of occupational injuries are homogeneous on each stratum, then, each study subject was selected by using simple random sampling technique that was used to select study participants. The numbers of samples from each stratum were determined using proportional allocation.

### 2.5. Data Collection and Data Quality Control

A semistructured, pretested questionnaire and observational checklist were used to collect data. The principal investigator, supervisor, and three data collectors participated in the data collection. The training was given for two days to the data collectors and the supervisor about the contents of data collection tools, questioning techniques, and ethical issues, and role play was used on how to fill the questionnaire. The questionnaire contains four components. The first deals with the sociodemographic characteristics of the hydropower construction workers. The second section probes about behavioral characteristics of participants which includes lifestyle questions such as chat chewing, and smoking. The third section is about working environment factors, i.e., job category, PPE utilization, working hour, and exposure to safety training among others. The last block of questions asks the outcome variable, and follow-up inquiries including occupational injury in the last 12 months, cause of injury, body part involved, and weather hospitalization were required. The questionnaire was pretested on 21 respondents working in Megech Dam construction at Gondar to identify potential problem areas, unanticipated interpretations, and cultural objections to any of the questions. Based on the pretest results, the questionnaire was adjusted. Content validity was assured by taking the pretest of questionnaires. Comments were collected from each participant, and the questionnaire was amended based on their suggestions. The internal consistency was analyzed by using Cronbach's *α* coefficient [17]. According to George [18], Cronbach's *α* coefficient value of >0.9 is taken as excellent, >0.8 as good, and >0.7 as acceptable. The Cronbach's *α* coefficient for the job satisfaction score, stress score, and overall score in the current study was 0.889, 0.936, and 0.930, respectively. The data collectors checked and corrected the collected data after completing the questionnaire before they left the site supervisors supervised the overall interview. The principal investigator and supervisor reviewed the completed questionnaires daily to ensure the completeness and consistency of the information collected. The respondents were interviewed separately to ensure privacy and to reduce bias due to discussion among themselves.

### 2.6. Operational Definitions

Occupational injury, the outcome variable of this study, was defined as any physical injury resulting from construction work in the past year before the study [12]. Job satisfaction was assessed by score measured using the job satisfaction scale as yes (32-40) and no (8-31) [19]. Job stress was assessed by score measured using the workplace stress scale as yes (16-40) and no (lower than or equal 15) [20]. Personal protective equipment (PPE) use was measured as the use of any specialized clothing or equipment by employees for protection against health and safety hazards. Workers were classified as those who used PPE when they were observed wearing the PPE that was necessary to be worn during a particular activity [12]. Study participant who drank at least five drinks of alcohol per week for men and two drinks per week for women for at least one year was taken as alcohol drinker [21]. A study subject who smoked one cigarette a day for at least one year was considered a smoker [21]. A khat chewer in the current study means someone who chews khat (a mild psychoactive substance) three times a week for at least one year [21].

### 2.7. Data Processing and Analysis

Data were entered using Epi-info version 7 and exported to SPSS version 20 for further analysis. For most variables, data were presented using frequency and percentage. Binary logistic regression analysis was used to choose variables for the multivariable binary logistic regression analysis, and variables with *P* value less than 0.2 during bivariable analysis were then analyzed by multivariable binary logistic regression for controlling the effect of confounders and variables which had a significant association with an occupational injury were identified based AOR with 95% CI and *P* < 0.05. We used the Hosmer and Lemeshow goodness-of-fit test to check model fitness. Variance inflation factor (VIF) is used to look at multicollinearity between variables.

## 3. Results

### 3.1. Sociodemographic Information

Four hundred and five study participants were included in this study with a 98.3% response rate. Three hundred ninety-three (97%) study subjects were male. The mean age of the study subjects was 29.1 ± 8.7 years. Only thirty-seven (9.1%) participants graduated from colleges or universities (Table 1).

### 3.2. Behavioral Factors

Fifty-eight (14.3%) of the study subjects smoke a cigarette, while 131 (32.3%) drink alcohol and 50 (12.3%) chew khat. Two hundred eighty-nine (71.4%) of the participants were satisfied with their current job (Table 2).

### 3.3. Working Environment Factors

Among the study participant majority, 331 (81.7%) attended workplace safety training. About 197 (48.6%) were temporary workers. Two hundred ten of the study subjects work on the night shift (Table 3).

### 3.4. Prevalence of Occupational Injury

**Figure 2:**
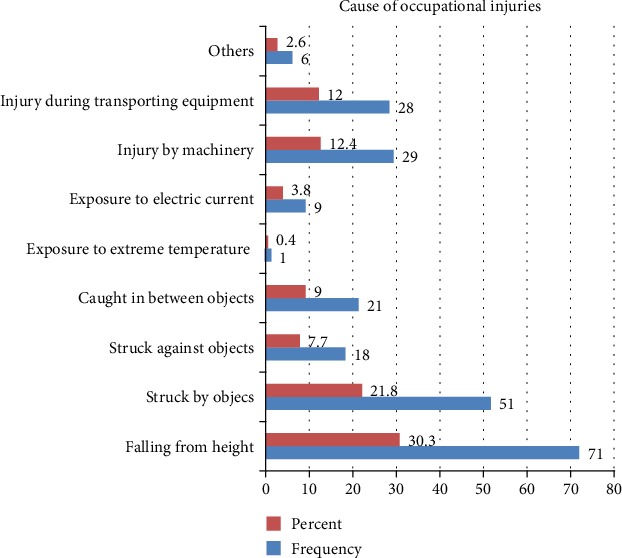
Cause of occupational injuries among Genale Dawa 3D hydropower dam construction workers South East Ethiopia, 2018.

From 405 participants included in this study, 234 (57.8%) with (95% CI (52.8, 62.7)) reported one or more occupational injuries. Among 234 injured workers, the four most common types of the cause of occupational injury reported were falling from a height (30.3%), struck by an object (21.6%), machinery (12.4%), and injury during transporting equipment (12%) (Figure 2).

Among the injured body parts of workers, hand (36.8%), lower leg (32.1%), and knee (11.1%) were the most affected body parts (Table 4).

**Figure 3:**
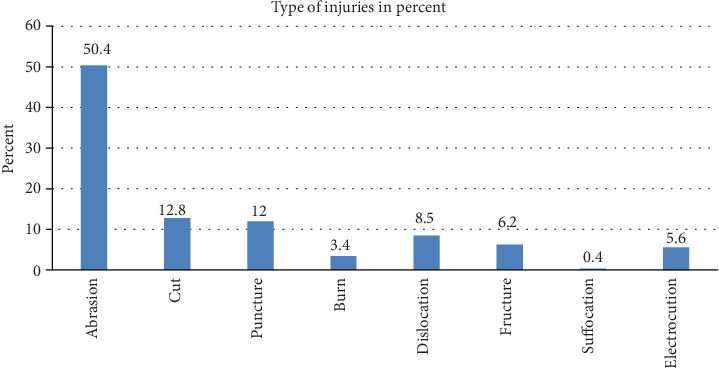
Type of occupational injuries among Genale Dawa 3D hydropower dam construction workers South East Ethiopia, 2018.

Regarding types of injuries, half of the injured workers faced 118 (50.4%) abrasion/laceration followed by 30 (12.4%) cut and 28 (12%) encountered puncture (Figure 3).

### 3.5. Factors Associated with Occupational Injuries

Age, alcohol consumption, chewing khat, job stress, job satisfaction, the working hour per week, and work shift were variables selected for the final model. The VIF test showed that for all variables, the result was below 3, the threshold for collinearity diagnostics. The occupational injury was significantly associated with age. The odds of having occupational injury were 2.63 times more likely to happen among participants aged 30-44 years as compared to study subjects aged 14-29 years with an odds ratio of 2.63 (AOR: 2.63, 95% CI (1.40, 4.94)).

This study showed that level of education was significantly associated with occupational injury. The odds of occupational injury were 3.64 times more likely to occur among illiterate study subjects as compared to study subjects who complete university/college education (AOR: 3.64, 95% CI (1.38, 9.56)).

The prevalence of occupational injury was 2.26 times more likely among participants who drink alcohol 217 (AOR: 2.26, 95% CI (1.22, 4.22)).

Study participants with job stress were 3.47 times more likely to be injured when compared to subjects who had not encountered job stress (AOR: 3.47, 95% CI (1.90, 6.35)).

Work shifts and working hours per week were significantly associated with occupational injury. In construction site, working over 48 hours per week raised the chance of getting an occupational injury by 2.4 times more likely than those who worked for ≤48 hours per week (AOR: 2.4, 95% CI (1.55, 3.73)). Study participants, who were working at night shift, were 2.65 times more likely to be injured as compared to those who worked at day shift (AOR: 2.65, 95% CI (1.18, 5.94)) (Table 5).

## 4. Discussion

The study results showed that the annual occupational injury prevalence of the dam construction site was 57.8% (95% CI (52.8, 62.7)). This result is higher than the prevalence reports of studies done on construction workers in Gondar [13], Addis Ababa [12], South West Ethiopia [22], and Egypt [9]. It is lower than the results of the other two studies done in Addis Ababa, Ethiopia [14, 15], and the other two studies conducted in Kenya [11, 22]. However, it is closer to the prevalence reported from a study done in Malaysia [23]. The discrepancy in the prevalence of occupational injuries might be because of differences in study populations, methods of data collection, workplace conditions and sample population, workplace safety standards and services, availability of personal protective equipment, and emphasis on workplace safety training. This high level of injury has an implication in the economy of the country, the families of the victims and the dam construction site.

The present study shows that the most frequent causes of occupational injuries were fall from a height, followed by struck by an object. This result is consistent with the study done in Kenya [11], Egypt [9], Malaysia [23], and Bangladesh [24]. The similarity may be because in the construction site falls from heights include crane falls, scaffolding falls, elevator shaft falls, and falls resulting from holes in flooring, and falling objects were a common cause of occupational injury. These may occur because of inadequate edge protection or from objects in the storage being poorly secured. Abrasions/lacerations and cutting were among the most common types of injuries according to this study.

The current study revealed that the hand and lower leg were the most injured parts of the body in construction work. This is supported by studies conducted on dam construction workers in Turkey [25] and Egypt [26], and different studies conducted in Ethiopia [13]. This could be because these body parts were the most active and low availability and use of PPE, lack of regular safety supervision, and training, however, inconsistent with findings from Bangladesh [27] and Ethiopia [13]. This might be because of adequate PPE availability and utilization in these studies.

Workers aged between 30 and 44 years were more likely injured than workers in the age group between 14 and 29 years old. This is contrary to studies conducted in Ethiopia [13] and Bangladesh [24] where there was no significant association between workers' age and occupational injury.

Illiterate workers were more likely injured than workers who complete university/college education. The result was similar to a study conducted on occupational injury among construction workers in Mombasa Kenya [28].

This study revealed that workers who were drinking alcohol were more likely injured than workers who were not drinking alcohol. A similar result was reported in a study conducted in Addis Ababa [16]. This might be because drinking alcohol is a proxy indicator of risk tolerance [29]. A high blood level of such substances during work will endanger both safety and efficiency, and cause an increased likelihood of mistakes, poor decision-making, and errors in judgment.

Perceived job stress was associated with occupational injuries that are workers who reported job stress were more likely to experience an injury than those who did not. This result is similar to a study done in Egypt [9]. This finding can be explained as job stress can cause physiological and psychological problems that may increase the risk of experiencing an occupational injury at work sites [1]. In the construction site, working over 48 hours per week increases the chance of getting an occupational injury. This is supported by studies conducted in Addis Ababa [14], South West Ethiopia [22], East Shoa, Ethiopia [30], and Egypt [9]. The fact could explain the reason fatigue associated with long hours of work may increase the likelihood of work-related injuries, and that long hour may result in injuries associated with breaching physical endurance limits. In addition, working over forty-eight hours per week will increase occupational injury as the injury is a function of exposure time [14]. Working over 48 hours fall under the International Labour Organization's excessively long hours category which particularly affects the workers. The longer working hour can be equated with tiredness increased likelihood of mistakes, not adhering standard operating procedures, poor decision-making, and errors in judgment and ultimately accidents [31]. Study participants working at night shift were more injured than those who were working at day shift. Working in shift causes a mismatch between the endogenous circadian timing system and the environmental synchronizers which affect human error precursors like sleepiness, chronic fatigue, and vigilance [32].

Finally, this study was not without limitations. Although much effort has been taken to reduce bias, social desirability and under or overreporting of annual occupational injury prevalence is expected as a limitation. Since the study is cross-sectional, it is not possible to infer a causal relationship and the recall bias due to the long time especially for minor injuries is expected.

## 5. Conclusions

The prevalence of occupational injuries among Genale Dawa 3D hydropower dam construction workers was high. Age, educational level, drinking alcohol, job stress, working hours per week, and work shift were factors significantly associated with occupational injuries. Among injured workers, abrasion and cutting by sharp material on the hand and lower leg occupied a high percentage. The common reasons for these injuries were falling from a height and struck by an object. Regular monitoring of substance abuse, avoiding overtime work, rotation of workers from one to another, and considering age and the educational status during employee recruitment may decrease the prevalence of occupational injuries among hydropower dam construction project workers.

## Figures and Tables

**Table 1:** Sociodemographic factors of Genale Dawa 3D hydropower dam construction workers South East 165 Ethiopia, 2018 (*n* = 405).

Variables		Frequency (*n*)	Percent (%)
Sex	Male	393	97
	Female	12	3

Age	14-29	276	68.1
	30-44	89	22
	≥45	40	9.9

Marital status	Single	207	51.1
	Married	155	38.3
	Divorced	30	7.4
	Widowed	4	1
	Separated	9	2.2

Education level	Illiterate	120	29.6
	Primary school(grade 1-8)	199	49.1
	Secondary school (grade 9-12)	49	12.1
	University/college	37	9.1

Monthly salary	1000-2000	133	32.8
	2001-4500	239	59
	>4500	33	8.1

**Table 2:** Behavioral factors of Genale Dawa 3D hydropower dam construction workers South East Ethiopia, 172 2018 (*n* = 405).

Variables	Frequency (*n*)	Percent (%)
Smoke cigarette	58	14.3
Drinking alcohol	131	32.3
Chewing khat	50	12.3
Sleeping problem	101	24.9
Job satisfaction	289	71.4
Job stress	100	24.7
Use PPE	295	72.8

**Table 3:** Working environment factors of Genale Dawa 3D hydropower dam construction project workers South East Ethiopia, 2018 (*n* = 405).

Variables		Frequency (*n*)	Percent (%)
Employment type	Temporary	197	48.6
	Permanent	208	51.4

Safety training	Yes	331	81.7
	No	74	18.3

Working hour per week	≤48 hours	266	65.7
	>48hours	139	34.3

Work shift	Day shift	195	48.1
	Night shift	210	51.9

Safety supervision	Yes	174	43
	No	231	57

Safety sign	Yes	242	59.8
	No	163	40.2

Manual handling activity	Yes	162	40
	No	243	60

Use vibrating material	Yes	119	29.4
	No	286	70.6

Job category	Daily labourers	208	51.6
	Electricians	95	23.5
	Site engineers	69	18.5
	Operator/drivers	27	6.7

**Table 4:** Summary of injured body part among Genale Dawa 3D hydropower dam construction workers South East Ethiopia, 2018 (*n* = 405).

Injured body part (*n* = 234)	Frequency (*n*)	Percent (%)
Eye	9	3.8
Teeth	7	3
Hand	86	36.8
Ear	7	3
Knee	26	11.1
Head	4	1.7
Upper arm	10	4.3
Lower arm	6	2.6
Lower leg	75	32.1
Chest	2	0.9
Others	2	0.9

**Table 5:** Summary of bivariate and multivariate analysis of factors on occupational injury among Genale Dawa 3D hydropower dam construction workers (*n* = 405).

Variables	Occupational injuries		COR (95% CI)	AOR (95% CI)
	No	Yes		
Age				
14-29	130	146	1.00	1.00
30-44	29	60	1.84 (1.12, 3.04)∗	2.63 (1.40, 4.94)∗∗
≥45	12	28	2.08 (1.02, 4.25)∗	2.00 (0.83, 4.84)
Educational status				
Illiterate	40	80	1.70 (0.80, 3.60)	3.64 (1.38, 9.56)∗∗
Primary school	94	105	0.95 (0.47, 1.92)	0.55 (0.18, 1.71)
Secondary school	20	29	1.23 (0.52, 2.92)	1.38 (0.49, 3.90)
University/college	17	20	1.00	1.00
Alcohols				
No	130	144	1.00	1.00
Yes	41	90	1.98 (1.28, 3.07)∗∗	2.26 (1.22,4.22)∗
Khat				
No	159	196	1.00	1.00
Yes	12	38	2.58 (1.30, 5.08)∗∗	0.74 (0.28, 1.90)
Work shift				
Day shift	94	101	1.00	1.00
Night shift	77	133	1.61 (1.08, 2.39)∗	2.65 (1.18, 5.94)∗
Work hours per week ≤48 hours	131	135	1.00	1.00
>48 hours	40	99	2.4 (1.55, 3.73)∗∗	2.48 (1.90, 6.64)∗∗
Job satisfaction				
No	39	77	1.66 (1.06, 2.60)∗	1.40 (0.75, 2.64)
Yes	132	157	1.00	
Job stress				
No	144	161	1.00	1.00
Yes	27	73	2.42 (1.47, 3.97)∗∗	3.47 (1.90, 6.35)∗∗

∗∗Significant at *P* value <0.01, ∗significant at *P* value <0.05.

## Data Availability

Data will be made available upon request of the primary author.

## Abbreviations

AOR: Adjusted odds ratio
CGGC: China Guzeuba Group Company
CI: Confidence interval
COR: Crude odds ratio
GDP: Gross domestic product
Km: Kilometer
MW: Megawatts
OSHA: Occupational safety and health administration
SPSS: Statistical package for social sciences
USD: United States Dollar
VIF: Variance inflation factor.
